# An Unexpected Connection: Potassium Limitation and Ammonium Toxicity in Yeast

**DOI:** 10.1371/journal.pbio.0040389

**Published:** 2006-10-17

**Authors:** Mary Hoff

Give the yeast *Saccharomyces cerevisiae* the right growing conditions and it multiplies like crazy—as any bread maker or beer brewer can testify. But deprive it of sufficient potassium, and it’s lucky to survive. Why? Since *S. cerevisiae* is a model organism for eukaryotes, the answer to that question could provide valuable insights into cellular processes of many organisms, including humans.

To learn how potassium limits growth in yeast, David C. Hess, David Botstein, and colleagues enlisted the assistance of DNA microarray analysis, a biochemical tool that allows scientists to identify which genes are active in a cell at any given time. What they found gave them a start: in *S. cerevisiae* cells grown in a potassium-limited medium, genes involved in a seemingly unrelated process—nitrogen metabolism—showed dramatically altered activity compared with unstressed cells, with genes repressed by products of nitrogen-compound breakdown becoming less active, and those whose products facilitate amino acid transport showing increased activity.

At first, that made about as much sense as discovering that every time you open your refrigerator the stock market drops. What could possibly be the connection? The altered gene activity pattern suggested an attempt to deal with a toxic influx of nitrogen within the cell, but nitrogen toxicity has been thought to be limited to multicellular organisms, with one-celled types easily able to keep the nutrient in balance by excreting excess through cell membrane channels. Could limited potassium upset that ability?

In search of an answer, the scientists looked at cells exposed to different ammonium and potassium levels. They found that in low-potassium but not high-potassium environments, cell numbers went down dramatically as ammonium concentration increased, suggesting that ammonium is indeed toxic to yeast when potassium is limited. A second test, in which they increased concentration of the nitrogen-rich amino acid asparagine rather than ammonium, confirmed that what they were seeing was not a general nitrogen effect, but one specific to ammonium. Further tests of other strains of *S. cerevisiae* confirmed that they were not dealing with a situation unique to a single quirky cell type.

If what they were seeing was indeed an adverse reaction to ammonium, the researchers predicted they should also see some sort of metabolic fingerprint of the yeast’s efforts to detoxify its environment. And they did. In collaboration with the Rabinowitz lab at Princeton, they used liquid chromatography tandem mass spectrometry to test the biochemical contents of medium in which ammonium-stressed yeast cells were grown. There, the researchers found high levels of amino acids—apparently the yeast equivalent of the urea we mammals excrete in urine to remove toxic nitrogen from our system.

**Figure:**
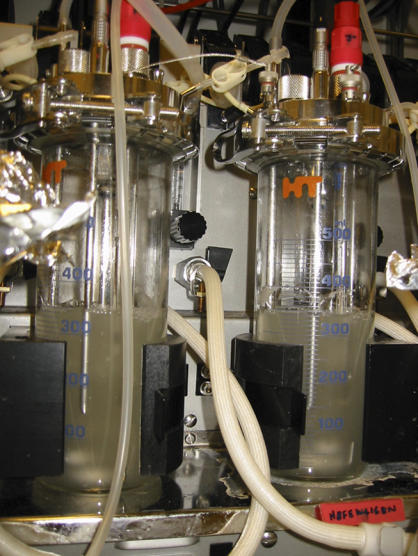
By applying systems-level biology to yeast cells growing in steady-state potassium limited chemostats (pictured above), the authors uncovered ammonium toxicity in yeast. (Image: Maitreya Dunham)

Having confirmed the presence of ammonium toxicity, the researchers next turned their attention to the issue of the mysterious connection with potassium concentration. Because potassium and nitrogen have similar chemical properties, they hypothesized that ammonium ions leak into cells through potassium channels when those channels are not otherwise occupied ushering potassium across the cell membrane. To test this, they engineered strains of *S. cerevisiae* in which ammonium influx into the cells could be increased without stimulating innate ammonium concentration regulatory mechanisms. Even in high-potassium environments, cells engineered to let in lots of ammonium showed greater mortality than those engineered to let in little, supporting the hypothesis that excess influx of ammonium is the root of the problem. Furthermore, the researchers found that in engineered cells in which ammonium transport across the cell membrane was high, growth was indeed limited even though potassium was not, and the cells excreted high levels of amino acid, mimicking the potassium-limited state.

The researchers concluded that *S. cerevisiae* does indeed experience ammonium toxicity under potassium-deprived conditions and that it uses a primitive detoxification system involving the production and excretion of amino acids in an attempt to deal with it. On a broader level, they demonstrated that systems biology techniques such as microarray analysis and mass spectrometry are valuable resources for discovering and exploring biochemical relationships and pathways that might otherwise remain masked in the normal workings of healthy cells. They hope in further studies to use these and other emerging tools to learn whether similar ammonium toxicity is also found in bacteria and to elucidate the mechanism behind *S. cerevisiae*’s amino acid–based detoxification system. For more on ammonium toxicity, see the related Primer (DOI: 10.1371/journal.pbio.0040388).

